# PAI-1 but Not PAI-2 Gene Deficiency Attenuates Ischemic Brain Injury After Experimental Stroke

**DOI:** 10.1007/s12975-018-0644-9

**Published:** 2018-07-05

**Authors:** Eva-Verena Griemert, Kirsten Recarte Pelz, Kristin Engelhard, Michael K. Schäfer, Serge C. Thal

**Affiliations:** Department of Anesthesiology, University Medical Center of the Johannes Gutenberg-University, Langenbeckstrasse 1, 55131 Mainz, Germany

**Keywords:** Stroke, Brain ischemia, Middle cerebral artery occlusion, Fibrinolysis, Plasminogen activator inhibitor-1, Plasminogen activator inhibitor-2

## Abstract

After stroke, secondary brain damage is influenced by the extent of fibrin clot formation. This is counteracted by the endogenous fibrinolysis. Of major interest are the key players of the fibrinolytic plasminogen activator system including the urokinase plasminogen activator (uPA), the tissue-type plasminogen activator (tPA), and their endogenous inhibitors plasminogen activator inhibitor 1 (PAI-1) and PAI-2. The role of PAI-1 in brain injury is well established, whereas the importance of PAI-2 is unknown at present. The objectives of the present were twofold: first, to characterize the time-dependent cerebral mRNA expression of the plasminogen activator system (PAS) after brain ischemia and second, to investigate the impact of PAI-1 and PAI-2 on brain infarct volume using gene-deficient mice. Adult *C57Bl/6J* mice were subjected to unilateral transient middle cerebral artery occlusion (MCAO) followed by reperfusion for 3, 24, 72, or 120 h. Quantitative PCR revealed that brain mRNA expression levels of the PAS components, and particularly of PAI-1 (237-fold) and PAI-2 (19-fold), peaked at 24 h after stroke. Accordingly, PAI-1 plasma activity was strongly increased. Brain infarct volume in TTC (2,3,5-triphenyltetrazolium chloride)-stained brain sections was significantly smaller 24 h after MCAO in PAI-1-deficient mice (− 31%), but not in PAI-2-deficient mice (− 6%). Thus, endogenous upregulation of PAI-1, but not of PAI-2, might contribute to increased brain damage after acute ischemic stroke. The present study therefore shows that PAI-2 is induced by brain ischemia, but does not play an important or relevant role for secondary brain damage after brain injury.

## Introduction

Stroke is the fourth leading cause of death and about 87% of the cases are caused by ischemic occlusion of a cerebral artery [1]. Therapeutic standard procedures are endovascular revascularization or systemic thrombolysis via tissue-type plasminogen activator (tPA) within 3 to 4.5 h after insult, but more than 90% of patients do not receive this treatment due to strict inclusion criteria or underutilization [2]. Therefore, characterizations of alternative neuroprotective interventions after stroke are necessary. In healthy vasculature, the fibrinolytic system is always active and protects the microcirculation against spontaneous clot formation. The most important physiological regulator of fibrinolysis is endogenous tPA that activates, just as urokinase PA (uPA), the proteolytic cleavage of plasminogen to plasmin. Plasmin, in turn, cleaves fibrin to soluble fibrin degradation products. Plasminogen activator inhibitors (PAIs) modulate fibrinolysis by inhibition of tPA and uPA. Particularly, PAI-1 is critically involved in the tight balance of pro- and anticoagulation and has been considered an acute-phase protein [3]. A variety of pro-inflammatory cytokines such as tumor necrosis factor alpha (TNFα), interleukin (IL)-1, and IL-6 as well as growth factors, hormones, and vasoactive peptides have been shown to stimulate PAI-1 production [4–7]. The inflammatory response after cerebral ischemia promotes fibrin clot formation by induction of PAI-1 and reduction of tPA plasma levels in stroke patients compared to controls [8]. Thus, high levels of PAI-1 impair the fibrinolytic system by binding to tPA and promoting stable fibrin clot formation, which obstruct micro vessels in the ischemic zone [9–12].

The key role of the plasminogen activator system in homeostasis of coagulation is attributed to PAI-1, although a marked upregulation of PAI-2 was reported after severe brain injury in human brain tissue and might also be a promising target [13]. Therefore, the present study investigates the role of both endogenous anti-fibrinolytic factors, PAI-1 and PAI-2, in an experimental stroke model of middle cerebral artery occlusion (MCAO). First, the time-dependent regulation of plasminogen activator system genes after stroke was examined by quantitative PCR (qPCR) and then the influence on the extent of ischemic brain injury was quantified in PAI-1- and PAI-2-deficient mice.

## Materials and Methods

### Animals

All animal procedures were performed in compliance with institutional guidelines of the Johannes Gutenberg-University Mainz, Germany. The Animal Ethics Committee of the Landesuntersuchungsamt Rheinland-Pfalz approved all experiments (protocol number 23177-07/G10-1-024). A total of 79 male *C57Bl/6J* (Charles River Laboratory, Sulzfeld, DE), *PAI-1* (Stock #002507), and *PAI-2* (Stock #007234) gene-deficient mice (JAX® Mice and Services, Jackson Laboratory, Bar Harbor, ME, USA), genetic background *C57Bl/6J*, between 8 and 12 weeks of age were investigated [14, 15]. Before and during the experiments, animals were kept in compliance with standard conditions (12-h day-night cycle, 60% humidity, 22 °C room temperature) and free access to food pellets and water.

### Transient Middle Cerebral Artery Occlusion and Experimental Groups

Mice were anesthetized by isoflurane via face mask (induction 4 vol%, maintenance 1.5 vol%). Body temperature was measured with rectal probe and maintained at 36.5 ± 0.5 °C using a feedback-controlled heating pad (Hugo Sachs, March-Hugstetten, DE). Brain ischemia was induced for 60 min by temporary MCAO using a silicone-coated 6–0 filament (6023; Doccol Corp., Redlands, CA, USA), while monitoring the CBF with a laser Doppler probe (PF 4001; Perimed, Järfälla, SE) essentially as described [16]. At the end of reperfusion time (after 3, 24, 72, or 120 h), the animals were deeply anesthetized, and the brains were carefully dissected after cervical dislocation.

The study includes parts of time course and gene deficiency investigations:

1. In total, 40 C57/Bl6J mice were randomized to MCAO with a reperfusion time of 3 h (*n* = 8, 6 surviving animals), 24 h (*n* = 7, 6 surviving animals), 72 h (*n* = 6), or 120 h (*n* = 11, 6 surviving animals) or to sham surgery (*n* = 2 per each time point; total *n* = 8 surviving animals). The mice of the sham group underwent the same procedures except the occlusion of the vessel.
2. In total, 39 wild-type C57/Bl6J (*n* = 10), PAI-1 (*n* = 10), and PAI-2 (*n* = 15) gene-deficient mice were randomized to MCAO (10 surviving animals per group) and C57/Bl6J to control group without surgery (*n* = 4 surviving animals). Brain ischemia was induced for 1 h by MCAO with 24 h of reperfusion.

### Assessment of Neurological Motor Skills

Neurological status was assessed by an investigator blinded to experimental groups. The modified neurologic severity score (NSS) was applied and comprised motor (muscle status, abnormal movement), sensory (visual, tactile, and proprioceptive), and reflex tests [17]. The authors Li et al. defined the severity of injury by the score graded on a scale of 0 to 14 (normal score 0, maximal deficit score 14). One point was awarded either for inability to perform, abnormal task performance, or lack of a tested reflex.

### Histological Evaluation and Tissue Sampling for Real-time qPCR

The dissected brains were cooled in 4 °C PBS for 5 min and afterwards cut in coronal 1-mm sections using a mouse brain matrix (Zivic Instruments, Pittsburgh, PA, USA). The tissue slices were immersed in 2,3,5-triphenyltetrazolium chloride (TTC) for 15 min at 37 °C and photographed (Leica; Wetzlar, DE). The volume of cerebral infarction was determined using DeltaPix Insight (DeltaPix, Maalov, DK). Both hemispheres were measured separately and the ratio of the non-ischemic part of the ipsilateral hemisphere minus the contralateral hemisphere corresponds with an edema-corrected infarct area. The sum of all infarct areas multiplied by the slice thickness is equivalent with edema-corrected infarct volume.

In the time course, samples (study 1) were collected from the left peri-ischemic area (penumbra) identified by the TTC staining. In the second set of experiments (study 2) with gene-deficient animals, brain samples were collected from the left upper quadrants of brain sections comprising the ischemic core and peri-ischemic tissue. Samples were snap-frozen in liquid nitrogen and stored at − 80 °C.

### Gene Expression Analysis

Total RNA was isolated using QIAzol Lysis Reagent (Qiagen, Hilden, DE) and RNA content was determined photometrically. Afterwards, RNA was reverse-transcribed into cDNA using the QuantiTect Reverse Transcription Kit (Qiagen). Quantitative RT-PCR analysis was performed with the LightCycler® 480 QPCR System (Roche, Grenzach-Wyhlen, DE; PAI-2), ABsolute™ Fast QPCR Mix (Thermo Scientific, Walldorf, DE; cyclophilin A [PPIA], PAI-1, IL-1β), or ABsolute™ Blue QPCR SYBR Green Mix (Thermo Scientific; TNFα, tPA, uPA). The quantities of the mRNAs were normalized to PPIA [18] and expressed as percentage of sham or native, respectively [19].

### Statistics

Statistical analysis was performed using GraphPad Prism 8 Software (GraphPad Software Inc., La Jolla, CA, USA). The Kruskal-Wallis test was used in each study and *p* values were adjusted for multiple comparisons (Dunn’s multiple comparisons test). The Welch’s test was applied when a pairwise comparison was needed. Results are presented as mean ± SEM. A *p* value < 0.05 was considered significant. As this is an explorative study, *p* values are given for descriptive reasons only. Descriptive *p* values are assigned as follows: ^*^p < 0.05, ^**^p < 0.01, ^***^p < 0.001.

## Results

### Inflammatory Parameters and Lesion Expansion Peaked at 24 to 72 h after MCAO

**Fig. 1:**
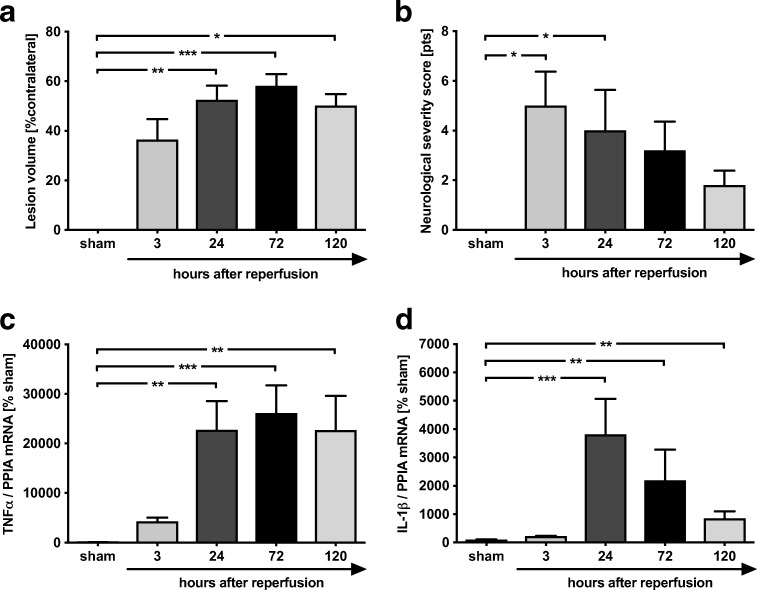
Time course of lesion expansion, neurological outcome, and regulation of inflammatory marker genes after MCAO. **a** The infarct volume increased over time with a peak of 58 ± 5% at 72 h (^***^p < 0.001 vs. sham). The lesion enlarged about 37 ± 8% at 3 h (*p* = ns vs. sham) to 53 ± 6% at 24 h (^**^p < 0.01 vs. sham) and declined at 120 h (50 ± 5%; ^*^p < 0.05 vs. sham). **b** The sensoric and reflex ability evaluated by the modified neurological severity score restored at 120 h after a decrease in impairment from 3 to 24 h (3 h, 5 ± 1.4 points, ^*^p < 0.05 vs. sham; 24 h, 4 ± 1.6 points, ^*^p < 0.05 vs. sham). TNFα and IL-1β as inflammatory marker genes were time-dependent regulated. **c** TNFα reached a peak at 72 h with a 261-fold increase compared to sham in tissue samples of peri-ischemic zone (^***^p < 0.001 vs. sham). **d** IL-1β peaked at 24 h with a 38-fold increase (^***^p < 0.001 vs. sham). Data are shown as mean ± SEM (*n* = 6 per group; sham: *n* = 8). Descriptive *p* values are assigned as follows: ^*^p < 0.05, ^**^p < 0.01, ^***^p < 0.001

At first, the time-dependent progression of infarct volume after MCAO was evaluated at reperfusion time of 3, 24, 72, or 120 h and compared to sham-operated mice. The edema-corrected infarct size increased over time with peak at 72 h (Fig. 1a). The lesion enlarged about 37 ± 8% at 3 h (*p* = ns vs. sham), 53 ± 6% at 24 h (*p* < 0.01 vs. sham) to 58 ± 5% at 72 h (*p* < 0.001 vs. sham), and 50 ± 5% at 120 h (*p* < 0.05 vs. sham). Neurological deficits were evaluated using a NSS adopted from Li et al. [17]. From 3 to 24 h reperfusion time, mice were severely compromised (3 h, 5 ± 1.4 points; 24 h, 4 ± 1.6 points; *p* < 0.05 vs. sham). At 72 and 120 h after MCAO, the NSS was not different compared to sham (Fig. 1b).

The time-dependent mRNA expression of inflammatory markers was determined in tissue collected from the peri-ischemic zone. TNFα mRNA expression peaked at 72 h after MCAO with a 261-fold increase (*p* < 0.001 vs. sham; Fig. 1c). The mRNA expression of IL-1β was upregulated at 24 h with a 38-fold increase compared to sham (*p* < 0.001 vs. sham; Fig. 1d) and declined over time. The results show a peak expression of inflammatory markers about 24 to 72 h, which is accompanied by the maximum of infarct volume caused by MCAO.

### Gene Expression of Plasminogen Activators and Their Inhibitors PAI-1 and PAI-2 Is Most Pronounced at 24 h After MCAO

**Fig. 2:**
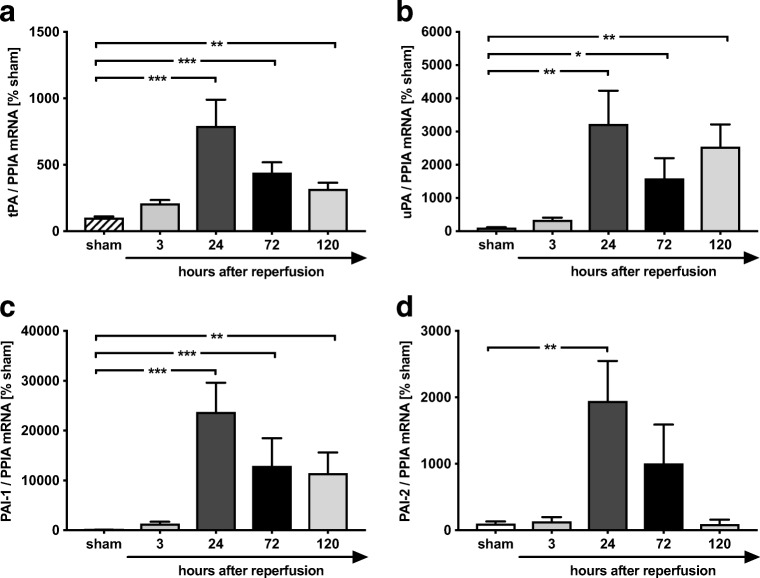
PAI-1 and PAI-2 is strongly regulated after MCAO. In tissue of ischemic injury, the expression of tPA and uPA peaked at 24 h (tPA ^***^p < 0.001 vs. sham (**a**); ^**^uPA *p* < 0.01 vs. sham (**b**)). Their opponents, PAI-1 and PAI-2, were massively upregulated after MCAO. **c** PAI-1 showed an increase at 3 h with a peak expression at 24 h (237-fold increase, ^***^p < 0.001 vs. sham). **d** Expression of PAI-2 showed a 19-fold increase at 24 h post insult (^**^p < 0.01 vs. sham). Data are shown as mean ± SEM (*n* = 6 per group; sham: *n* = 2 per each group). Descriptive *p* values are assigned as follows: ^*^p < 0.05, ^**^p < 0.01, ^***^p < 0.001

In a next step, we determined mRNA expression levels of the plasminogen activator system. The time-dependent effects after MCAO on mRNA expression of the plasminogen activator system components tPA, uPA, PAI-1, and PAI-2 were quantified in peri-ischemic tissue at 3, 24, 72, and 120 h after reperfusion and were compared to sham surgery. The mRNA expression levels of plasminogen activators peaked at 24 h post insult (tPA, *p* < 0.001 vs. sham; uPA, *p* < 0.01 vs. sham; Fig. 2a, b). The uPA expression showed a second peak at 120 h post injury (*p* < 0.01 vs. sham; Fig. 2b). The mRNA expression of PAI-1, the main inhibitor of plasminogen activators, was already upregulated at 3 h with 237-fold increase at 24 h after MCAO (*p* < 0.001 vs. sham; Fig. 2c). Also, PAI-2 mRNA expression increased 19-fold at 24 h post insult (*p* < 0.01 vs. sham; Fig. 2d) and decreased to baseline values over time. In summary, the data show a strong upregulation of the plasminogen activators and their inhibitors PAI-1 and PAI-2 at mRNA level in response to MCAO. The mRNA expression levels of PAI-1 are more intensively regulated compared to plasminogen activators and show a peak expression at 24 to 72 h post injury, along with the maximum of infarct size.

### Markers of Plasminogen Activator and Inflammation System Were Not Affected by PAI Gene Deficiency After MCAO

The following experiments were conducted to explore whether PAI gene deficiency interferes with the mRNA expression of plasminogen activator system genes or inflammatory marker genes. The mRNA expression of PAIs was strongly increased in wild types about 50-fold for PAI-1 mRNA (*p* < 0.01 vs. native) and 33-fold for PAI-2 mRNA (*p* < 0.05 vs. native). PAI-1 was also upregulated in PAI-2-deficient mice after 24-h reperfusion (PAI-2^−/−^ 53-fold, *p* < 0.05 vs. native). No PAI-1 expression was detectable in PAI-1-deficient mice. Also, PAI-2 expression was similar in PAI-1-deficient and wild-type mice (PAI-1^−/−^ 32-fold, *p* < 0.001 vs. native; PAI-1/PAI-2^+/+^ 33-fold, *p* < 0.05 vs native). Therefore, the gene function of PAI-1 and PAI-2 seems to be independently regulated of each other and compensatory effects were absent in PAI-deficient mice.

**Fig. 3:**
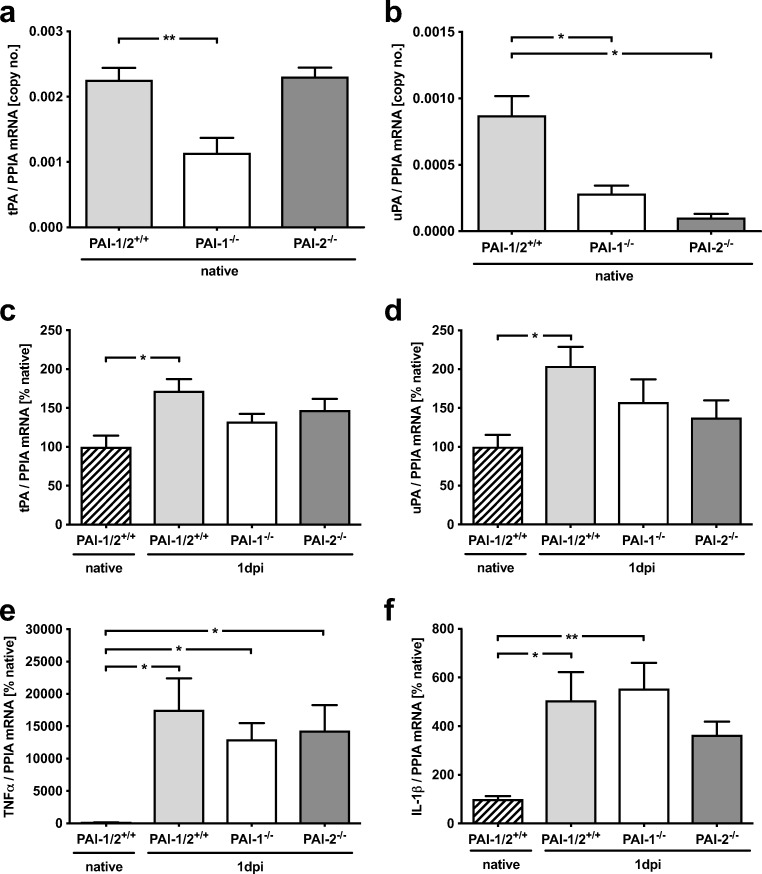
mRNA regulation of fibrinolytic and inflammatory marker genes in PAI-1 and PAI-2-deficient mice after MCAO. **a**, **b** Comparing the expression of tPA and uPA in native wild-type animals compared to PAI-1 and PAI-2-deficient mice. PAI-1-deficient mice show significantly lower tPA and uPA expression levels compared to wild-type mice, whereas PAI-2-deficient mice demonstrate only significantly lower uPA levels in the brain. **c**, **d** Modulation of tPA and uPA was similar to the time course study and without differences between groups at 1 day past injury (1 dpi). **e**, **f** Inflammatory parameters were upregulated over time without differences between groups. Data are shown as mean ± SEM (*n* = 10 per group; native: *n* = 4). Descriptive *p* values are assigned as follows: ^*^p < 0.05, ^**^p < 0.01, ^***^p < 0.001 using the Kruskal-Wallis test; ^#^*p* < 0.05, ^##^*p* < 0.01, ^###^*p* < 0.001 using Welch’s test

To determine the influence of PAI-1 and PAI-2 deficiency on tPA and uPA expression, brain tissue samples from native animals were quantified. PAI-1-deficient mice showed significantly lower tPA (Fig. 3a) and uPA (Fig. 3b) expression levels compared to wild-type mice, whereas PAI-2-deficient mice demonstrated significantly lower uPA levels in the brain. The data suggest that PAI-1 deficiency influences plasminogen activator system in the healthy conditions, whereas PAI-2 deficiency has only an influence on the uPA system. mRNA expression in peri-ischemic tissue at 24 h after reperfusion showed a significantly upregulated tPA and uPA expression in injured wild types compared to native mice without any differences between the gene-deficient mice and injured wild-type mice (tPA: *p* < 0.05 vs. native, Fig. 3c; uPA: *p* < 0.05 vs. native, Fig. 3d). Interestingly, tPA expression was not upregulated in PAI-2-deficient animals (Fig. 3c**)**. Moreover, no differences between insult groups were detectable in the mRNA expression of the inflammatory markers TNFα and IL-1β (Fig. 3e, f). Taken together, the PAI-1 and PAI-2 mRNA expression was markedly upregulated after MCAO compared to sham mice.

### Infarct Volume Was Reduced in PAI-1, but Not in PAI-2-Deficient Mice

**Fig. 4:**
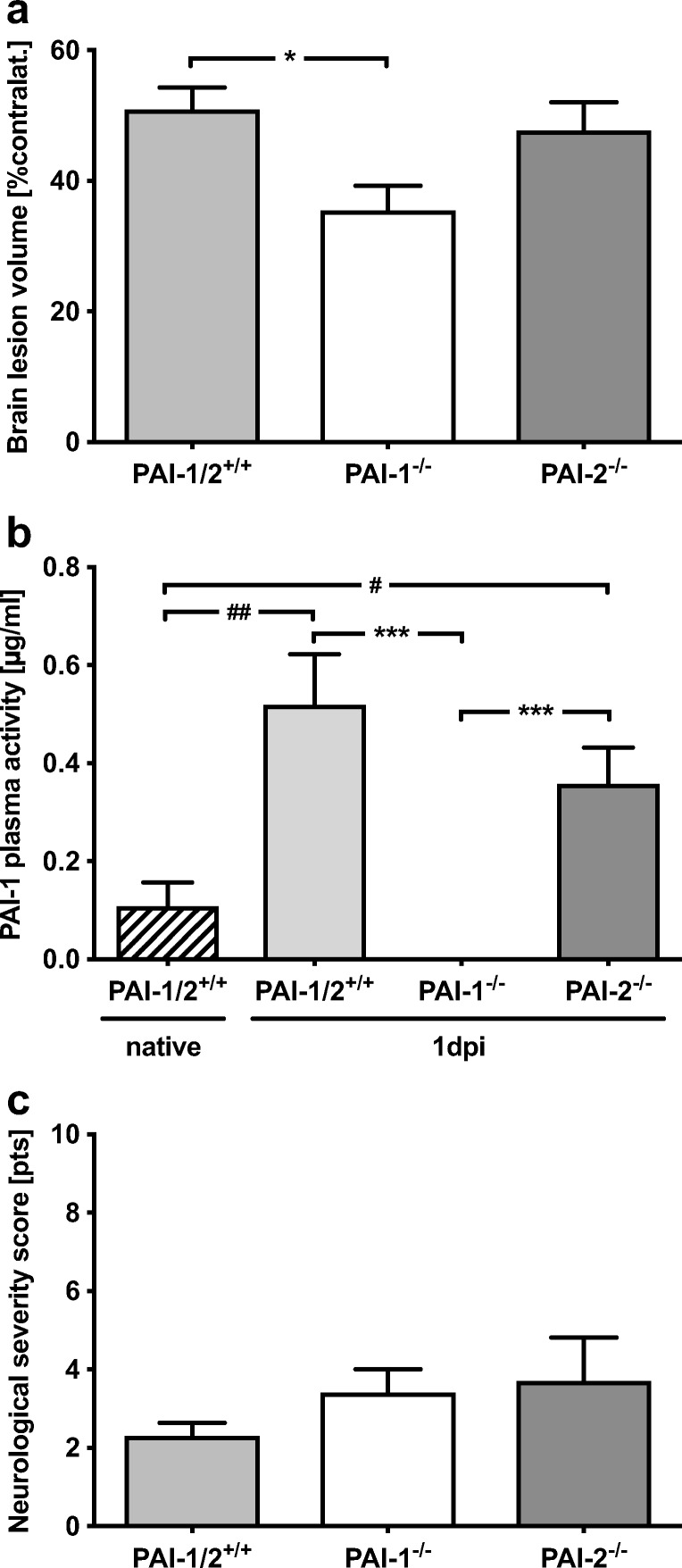
Reduced infarct volume in PAI-1-deficient mice. **a** The infarct volumes were reduced in PAI-1-deficient mice by 31% compared to wild-type mice 24 h after insult (PAI-1^−/−^ 35 ± 11% of contralateral hemisphere, ^*^p < 0.05; PAI-1^+/+^/PAI-2^+/+^ 51 ± 10% of contralateral hemisphere). PAI-2 deficiency did not influence brain injury (48 ± 13% of contralateral hemisphere). **b** The PAI-1 plasma activity was undetectable in PAI-1-deficient mice but increased over time without differences between wild-type or PAI-2-deficient mice (PAI-1^+/+^/PAI-2^+/+^ 5-fold increase, ^##^*p* < 0.01 vs. native; PAI-2^−/−^ 3.5-fold increase, ^#^*p* < 0.05 vs. native). **c** The neurological severity score showed no differences between groups. Data are shown as mean ± SEM (*n* = 10 per group; native *n* = 4). Descriptive *p* values are assigned as follows: ^*^p < 0.05, ^**^p < 0.01, ^***^p < 0.001 using the Kruskal-Wallis test; ^#^*p* < 0.05, ^##^*p* < 0.01, ^###^*p* < 0.001 using Welch’s test

To investigate the role of PAI-1 and PAI-2 in brain tissue injury, PAI-1- and PAI-2-deficient mice and wild-type animals were subjected to MCAO and brain damage was determined at 24 h after insult. The lesion volume in wild-type mice was 51 ± 10% of contralateral hemisphere (Fig. 4a). In PAI-1-deficient mice, the lesion volume was significantly reduced by 31% compared to wild-type mice (35 ± 11% of contralateral hemisphere, *p* < 0.05 vs. PAI-1^+/+^/PAI-2^+/+^). In contrast to PAI-1-deficient mice, PAI-2 deficiency did not influence the extent of brain damage (48 ± 13% of contralateral hemisphere). As expected, PAI-1 plasma activity was not detectable in PAI-1-deficient, but in wild-type and PAI-2-deficient mice (Fig. 4b). The plasma activity showed no different regulation between wild-type and PAI-2-deficient mice (WT 5-fold increase, *p* < 0.01 vs. native; PAI-2^−/−^ 3.5-fold increase, *p* < 0.05 vs. native). The neurocognitive function determined with a neurological severity score (NSS) did not differ between groups at 24 h after MCAO (Fig. 4c).

## Discussion

After ischemic stroke, a tight balance of the endogenous fibrinolytic system is of immense relevance to avoid further brain damage due to hypercoagulation. Dysregulation of plasminogen activators and their inhibitors, mainly tPA and PAI-1, can cause obstruction of microvessels or excessive bleeding [20]. The present study focused on the questions whether the plasminogen activator system is influenced by stroke and how this interferes with the extent of brain injury. The results showed a peak of PAI-1 and PAI-2 mRNA expression levels in the brain and increased PAI-1 plasma activity at 24 h after MCAO. At the same time, infarct volume was reduced in PAI-1- but not in PAI-2-deficient mice. The results confirm the role of PAI-1 after ischemic stroke and provide first data on the limited role of PAI-2 in brain injury progression following experimental stroke.

In the present study, the mRNA expression profiles of tPA and uPA as well as PAI-1 and PAI-2 were described for the first time in detail after MCAO. The results are important because they may help to find a therapeutic window for interventions in the acute phase of stroke. A major finding of the present study was the massive upregulation after stroke of PAI-1 compared to the other players such as tPA, suggesting a disbalance of the PA system and their inhibitors on the mRNA level with a shift towards an anti-fibrinolytic state about 24 to 72 h after MCAO. This was not only present on the mRNA level, but also reflected by an increased plasma activity of PAI-1 after MCAO. Consistently, a clinical prospective incident case-control study confirmed that an increase in PAI-1 plasma levels was present in the acute phase of stroke and that high plasma levels of PAI-1 enhance in turn the risk of stroke [21]. Moreover, this increase was associated with poor neurological outcome and potentially linked to higher mortality rates [9, 22]. In the present study, we also observed a correlation between neurological deficits and fibrinolysis after stroke. The highest neuroscores, representing severe neurological deficits, were found between 3 and 72 h after insult which is coincident with the mRNA peak expression of inflammation and PAI system parameters. At 120 h post MCAO, the mRNA expression level of most parameters was reduced, and the neurological performance recovered. These data suggest that elevated mRNA and most likely also PAI-1 protein levels may counteract the fibrinolytic effect of plasminogen activators, prevent re-opening of the occluded microvessels, and thereby increase brain damage.

After we have shown that PAI-1 and to a lesser extent PAI-2 mRNA expression is increased in brain tissue after MCAO, we wanted to characterize the influence of the PAI system on brain damage after stroke using PAI-1- and PAI-2 gene-deficient mice. In a first step, we show that compared to wild-type mice in these gene-deficient mice, no compensatory regulation of mRNA expression levels for inflammatory (TNFα and IL-1β) and fibrinolytic (tPA and uPA) markers occurred 24 h after MCAO, which was in concordance with data of a systemic inflammation model in PAI-1 deficiency [23]. In the present study, the infarct volume was reduced 24 h after MCAO in PAI-1-deficient mice. This was not associated with changes of the inflammation or plasminogen activator systems, as these parameters were not different between groups (wild type, PAI-1^−/−^, PAI-2^−/−^). Therefore, it is likely that the anti-fibrinolytic effect of PAI-1 directly deteriorates outcome after MCAO, possibly by inhibiting the lysis of fibrin clots in microvessels of the penumbra. This hypothesis is supported by several studies. After the MCAO in rats using a fibrin deposit model in microvessels, PAI-1-dependent suppression of fibrinolysis correlated with impaired cerebral perfusion [24]. In a stroke model of transgenic overexpression of PAI-1, recanalization after MCA thrombosis was most likely delayed by inhibition of fibrinolysis. This was considered as the intravascular pathomechanism leading to increased brain injury [25]. Vice versa, PAI-1 gene-deficient mice developed less venous thrombosis after endotoxin injection in the footpath as a thrombosis model [26]. This effect was independent of changes of inflammation. Moreover, attenuation of fibrin deposition led to an improved neurological outcome in an ischemic stroke model [10]. Multiple studies proved in various thrombosis models an antithrombotic effect of PAI-1 inhibition without negative side effects on hemostasis or platelet function [27]. However, there are contradictory findings in rodent stroke models showing a detrimental effect of PAI-1 inhibition after brain injury [28]. Most likely, the stroke model (with or without reperfusion) is relevant for fibrin clot formation in micro vessels and the beneficial or detrimental effect of PAI-1 [12, 25]. Lysis of these fibrin clots is most likely the major protective mechanism of PAI-1 inhibition. This protective effect is more pronounced in a MCAO model with reperfusion [12, 25]. Therefore, it is assumed that reduced infarct size in PAI-1-deficient mice is caused by a sufficient intravascular tPA-mediated fibrinolysis that is not blocked by an excessive PAI-1 action. Recently, targeting PAI-1 by means of a monoclonal antibody showed beneficial effects after ischemic stroke [10, 29]. Interestingly, neurofunctional deficits were not improved in PAI-1-deficient animals. Similarly, recent data did not also show reduced brain damage, but no improved neurofunction in animals treated with PAI-1 antibodies [10].

In contrast to PAI-1, PAI-2 expression is usually low or not detectible. Under normal conditions, PAI-2 is detectable in keratinocytes, macrophages, activated monocytes, placenta, and also cells of neuronal origin [6, 30]. PAI-2 signal is also present in microglia and vascular endothelial cells in human brains and increased levels were shown in injured human brains [13, 31]. In endothelial cells, PAI-2 expression is modulated by lipopolysaccharide, phorbol ester, TNFα, IL-1, and angiotensin II [6]. The role of PAI-2 in fibrinolysis is not well established. Probably the first report on PAI-2-dependent effect on thrombus formation was the work by Siefert et al. [32]. In a deep vein thrombosis mouse model, PAI-2-deficient mice exhibited no thrombus resolution at day 2, 4, or 8, but an enhanced resolution at day 12, which was attributed to increased uPA activity [32]. In the present study, PAI-2 mRNA expression is strongly upregulated. Although less pronounced compared to the PAI-1 expression, the PAI-2 expression pattern suggests an important role for secondary brain damage. Surprisingly, PAI-2 knockout did not influence the extent of brain damage or neurofunction deficits. In addition, post-ischemic cerebral inflammation was also not significantly different between wild-type and PAI-2-deficient animals. Native PAI-2-deficient animals demonstrated wild-type tPA levels. Interestingly, post-ischemic upregulation of tPA was not present in PAI-2-deficient animals. This may indicate that lack of PAI-2 is not compensated by upregulation of uPA or tPA. The authors cannot rule out that other factors are regulated upon PAI-2 deficiency, which contribute to secondary lesion formation. Overall, the present data suggest that PAI-2 seems to play only a minor role after cerebral ischemia.

The present study is limited due to the use of 2–3-month-old male mice. We cannot rule out that experiments with older or female mice may lead to additional findings with respect to PAI-1 and PAI-2 deficiency. The observation period of 24 h is suitable to focus on the acute phase with peak expression of PAI-1 and PAI-2. PAI-1 and PAI-2 were upregulated in samples from patients up to 70 h after ischemic stroke [13]. However, the present study does not include long-term observation data. Therefore, it cannot be ruled out that delayed processes during recovery and regeneration after ischemic stroke are also affected by PAI-1 or PAI-2. Putative delayed effects require further investigations. The role of PAI-1 and the protective effect of PAI-1 inhibition are well established in other studies with survival time points up to 24 h [10, 25]. PAI-2 on the other hand failed to show any effect in the present study and it appears to be unlikely that extending the observation period would show a beneficial effect of the PAI-2 knockout.

In conclusion, endogenous upregulation of PAI-1, but not of PAI-2, might contribute to increased brain damage after acute ischemic stroke. The present data therefore show that PAI-2 is strongly induced by brain ischemia, but the present study provides solid data that PAI-2 does not play an important or relevant role for secondary brain damage after acute ischemic brain injury.
